# Application of ^18^F-FDGPET/CT in primary isolated pulmonary solitary fibrous tumor

**DOI:** 10.3389/fmed.2025.1552628

**Published:** 2025-04-17

**Authors:** Mingyan Shao, Sisi Fan, Wanling Qi

**Affiliations:** ^1^Department of Nuclear Medicine, Jiangxi Provincial People’s Hospital, The First Affiliated Hospital of Nanchang Medical College, Nanchang, Jiangxi, China; ^2^Department of Pathology, Jiangxi Provincial People’s Hospital, The First Affiliated Hospital of Nanchang Medical College, Nanchang, China

**Keywords:** solitary fibrous tumor, pulmonary, metastasis, CT, ^18^F-FDG PET/CT

## Abstract

**Objective:**

Pulmonary solitary fibrous tumors (SFTs) represent a rare clinical entity, with malignant variants demonstrating particularly aggressive behavior and metastatic potential. The diagnostic challenge in early-stage disease underscores the need for improved detection methods. This study evaluates the diagnostic utility of ^18^F-FDG PET/CT in distinguishing benign from malignant pulmonary SFTs and assesses its role in treatment response monitoring.

**Methods:**

We performed a retrospective analysis of clinical characteristics and imaging findings in four histologically confirmed pulmonary SFT cases evaluated with ^18^F-FDG PET/CT at Jiangxi Provincial People’s Hospital (2020–2024).

**Results:**

The cohort exhibited heterogeneous clinical presentations: three patients reported chest tightness with pain, one had non-painful chest tightness, and two presented with concomitant cough. Notably, no cases demonstrated hemoptysis, productive sputum, or fever. Contrast-enhanced CT initially suggested malignancy in three cases and benign pathology in one. PET/CT revealed two cases with intense FDG avidity (preoperatively classified as malignant—one with peritoneal metastases) and two with minimal uptake (classified as benign). Histopathological confirmation of all surgical specimens established PET/CT’s 100% diagnostic accuracy for benign/malignant differentiation, compared to only 50% accuracy for contrast-enhanced CT.

**Conclusion:**

^18^F-FDG PET/CT provides clinically valuable discrimination between benign and malignant pulmonary SFTs, while offering additional benefits in disease staging, biopsy guidance, treatment response assessment, and post-therapeutic surveillance.

## 1 Introduction

Solitary fibrous tumors (SFTs) represent a rare mesenchymal neoplasm originating from CD34-positive dendritic stromal cells (1). Characterized by their fibroblastic and myofibroblastic differentiation potential, these tumors demonstrate a spectrum of biological behavior. While most SFTs follow a benign clinical course, approximately 10–20% exhibit malignant transformation (2). Malignant variants display ubiquitous anatomical distribution without gender predilection and frequently present with nonspecific symptoms, contributing to diagnostic challenges (3). The current therapeutic paradigm for malignant SFTs prioritizes wide local excision as the cornerstone of management. In metastatic disease, systemic chemotherapy remains the primary treatment modality (4). However, robust data regarding chemotherapeutic response assessment are notably lacking in the literature, with existing evidence limited to isolated case reports. This knowledge gap significantly constrains clinical decision-making for malignant pulmonary SFTs. In this context, we present ^18^F-FDG PET/CT findings from four cases of malignant pulmonary SFTs, accompanied by comprehensive clinicopathological correlation. Through systematic analysis of imaging characteristics and treatment outcomes, supplemented by literature review, this study aims to improve diagnostic recognition and inform therapeutic strategies for this rare entity.

## 2 Methods and materials

### 2.1 Patients and materials

In this retrospective study, we evaluated the clinical features and imaging manifestations of four cases of solitary fibrous tumors diagnosed using ^18^F-FDG PET/CT at the First Affiliated Hospital of Nanchang Medical College from 2020 to 2024.

### 2.2 Diagnostic approach

All patients underwent preoperative ^18^F-FDG PET/CT scans. The scans were reviewed blindly by one attending physician and one chief physician specializing in PET/CT. In cases of disagreement, a collective decision was made by the departmental physicians. All four patients eventually underwent surgical resection, and the postoperative pathological typing was determined by discussion between one chief pathologist and one attending pathologist.

Informed consent for the anonymous use of clinical data and imaging for publication purposes was provided by all patients. Given the retrospective nature of this study, the institutional review board waived the requirement for approval.

## 3 Results

### 3.1 Case 1

**Figure 1:**
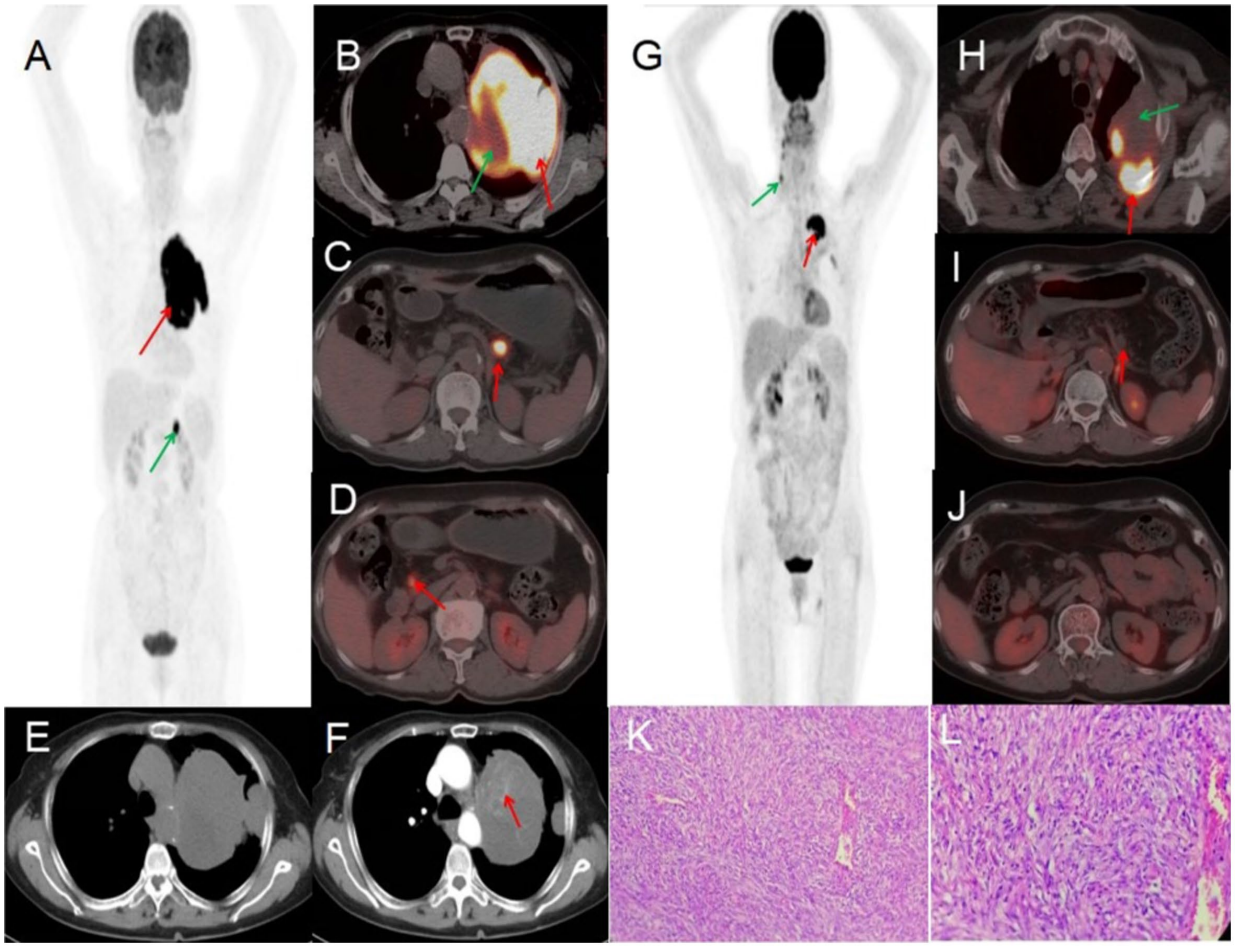
Female, 70 years old, malignant pulmonary solitary fibrous tumors. **(A)** Whole body MIP. **(B–D)** Axial fusion. **(E)** Axial, non-contrast-enhanced CT. **(F)** Axial, enhanced CT-arterial phase. ^18^F-FDG PET/CT imaging findings: a large soft tissue mass was identified in the upper lobe of the left lung, exhibiting heterogeneous density with multiple patchy hypodense areas and ill-defined margins. The mass showed loss of the fat plane adjacent to the mediastinum, encasement of the left upper pulmonary artery, and associated pleural thickening and adhesions. On contrast-enhanced CT, the lesion demonstrated marked heterogeneous enhancement in the arterial phase, with internal septations and multiple enhancing vascular structures (arrow, **F**). The mass measured 88 × 108 mm in maximum dimensions and exhibited intensely increased metabolic activity, with a maximum standardized uptake value (SUV_max_) of 23.2 (red arrow, **B**). No FDG uptake was observed in the central hypodense necrotic regions (green arrow, **B**). Additionally, two hypermetabolic nodules were noted in the pancreatic head and retropancreatic region (arrows, **C,D**), demonstrating an SUV_max_ of 13.2. **(G)** Whole body MIP. **(H–J)** Axial fusion. **(K)** H-E ×200. **(L)** H-E ×400. Follow-up ^18^F-FDG PET/CT imaging (post-chemotherapy). The previously documented solitary fibrous tumor of the lung demonstrated significant size reduction following one year of chemotherapy. Imaging findings: a heterogeneous, ill-defined mass (approx. 50 × 73 mm in maximal cross-section) persists in the left upper lobe, with adjacent pleural thickening and adhesions. Metabolic activity has markedly decreased, with residual FDG uptake localized to the posteromedial margin (SUV_max_ 10.8, red arrow, **H**). No significant metabolic progression was observed in the known intraperitoneal metastatic lesions (retropancreatic and pancreatic head regions, red arrows, **I,J**). A focal FDG-avid focus in the right neck was deemed non-specific (green arrow, **G**). Pathological correlation (microscopic features): tumor cells display mild to moderate atypia, with round to oval morphology and a predominant spindle-cell composition. The stroma exhibits collagen-rich fibrosis, focal myxoid degeneration, and scattered areas of mitotic activity and hypercellularity **(K,L)**.

A 70-year-old female presented with symptoms of chest tightness, discomfort, and occasional non-productive cough on March 13, 2022. She reported mild chest pain but denied shortness of breath, chills, fever, dizziness, headache, abdominal pain, or distension. The patient was admitted to Jiangxi Provincial People’s Hospital for further evaluation and management. Since symptom onset, she maintained normal mental status, appetite, sleep patterns, and bowel and bladder function without significant weight changes. The patient had no history of chronic disease and a history of other types of tumor. Tumor markers were within normal limits, except for slightly elevated levels of CYFRA21-1 3.50 (0–3.3) ng/mL, NSE 22.77 (0–16.3) ng/mL, and CEA6.98 (0–6.5) ng/mL. Lung CT revealed a large, ill-defined soft tissue density mass in the left upper lung with heterogeneous internal density, including areas of low-density necrosis, and irregular enhancement on contrast-enhanced scan. Abdominal CT showed nodular shadows posterior to the pancreatic body and adjacent to the pancreatic head, demonstrating mild to moderate enhancement on contrast imaging. CT findings were consistent with a malignant tumor. ^18^F-FDG PET/CT demonstrated a large, heterogeneously dense soft tissue mass in the left upper lobe with multiple patchy low-density areas, indistinct margins, loss of fat planes between the mass and mediastinum, encasement of the left upper pulmonary artery, and adjacent pleural thickening and adhesion. The mass measured 88 × 108 mm with markedly increased FDG uptake (SUV_max_ 23.2), while the necrotic regions showed no FDG avidity. Two pancreatic nodules—near the head and posterior to the body of the pancreas—also exhibited elevated FDG uptake (SUV_max_ 13.2). Preoperative PET/CT suggested pulmonary malignancy with intraperitoneal metastasis. A CT-guided needle biopsy was performed, with histopathology confirming a solitary fibrous tumor (SFT) of the left upper lung, characterized by high cellularity and active proliferation. Due to the large mass and abdominal metastasis, medical treatment was initiated after extensive consultation and comprehensive evaluation within the hospital. The patient was treated with bevacizumab 400 mg on day 1 and temozolomide 220 mg on days 1–5. After one treatment in this cycle, patients were treated with cindilizumab (200 mg) + alotinib (12 mg on days 1 to 14), supplemented with stomach protection, antiemetic, and immune correction therapy. After one year of treatment, the patient’s response was satisfactory: PET/CT scan demonstrated a significant decrease in the volume of the solitary fibrous tumor in the lungs following chemotherapy, along with a notable reduction in FDG uptake in the masses. No significant increase in FDG uptake was observed in intraperitoneal metastatic lesions located behind the pancreatic body or near the head of the pancreas, indicating that the active part of the lung tumor had been inactivated, and the activity of the intra-abdominal metastases had been completely suppressed. After multidisciplinary consultation, because the patient had signs of distant metastasis and a large primary tumor, she performed intraoperative lymph node dissection while surgical resection of the mass. Postoperative pathology revealed that a primary lung malignant solitary fibrous tumor was suspected, with no tumor involvement at the incisal margin of the bronchus. Regional lymph nodes showed no evidence of tumor metastasis in nine lymph nodes examined (two of which exhibited necrotizing granulomatous inflammation). The vascular tissue (artery wall) was examined and found to have fibrous tissue hyperplasia with glass change. The patient received comprehensive anti-tumor treatment post-surgery (see Figure 1).

### 3.2 Case 2

**Figure 2:**
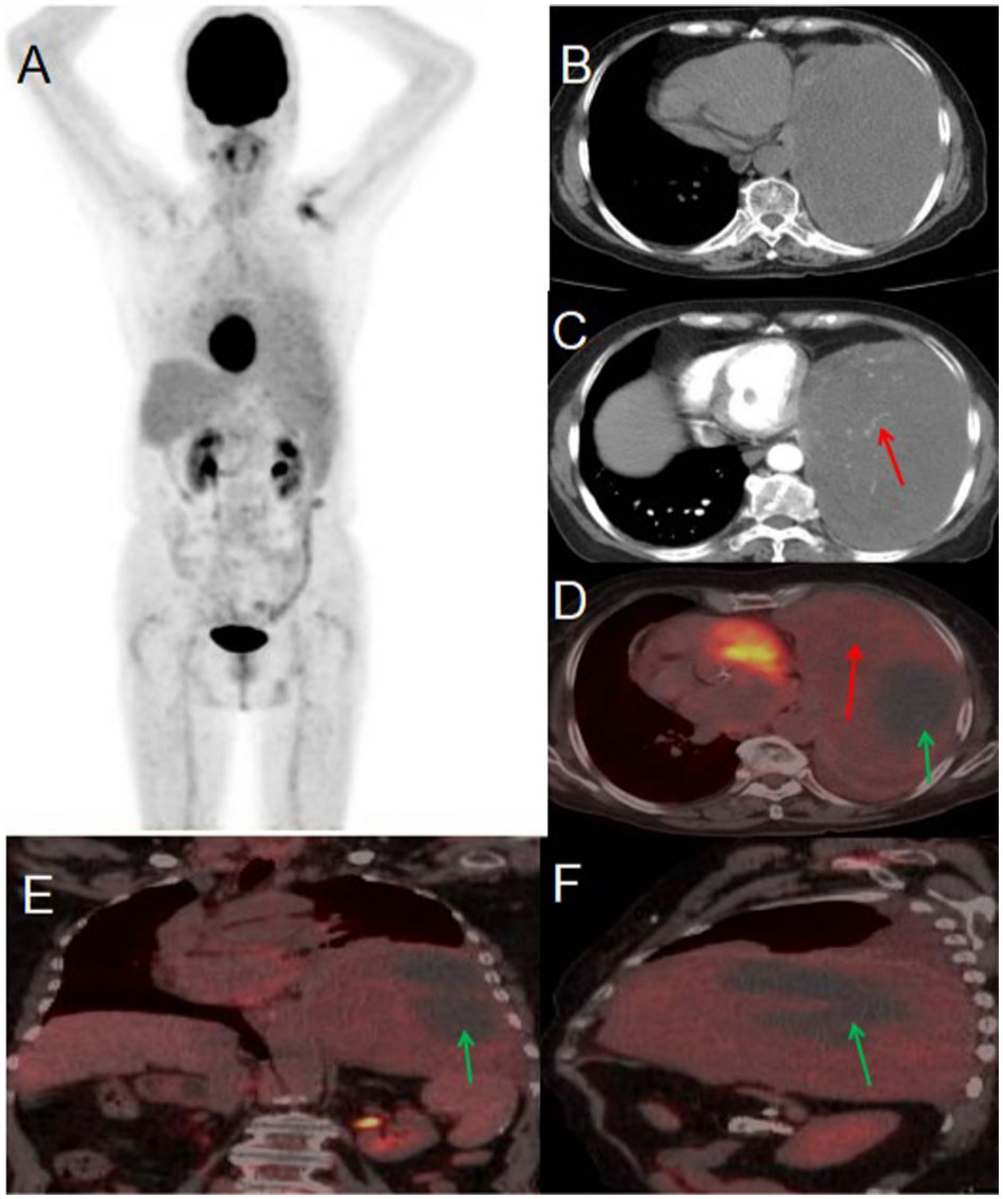
Male, 80 years old, benign primary pulmonary solitary fibrous tumors. **(A)** Whole body MIP. **(B)** Axial, non-contrast-enhanced CT. **(C)** Axial, enhanced CT-arterial phase. **(D)** Axial fusion. **(E)** Coronal fusion. **(F)** Sagittal fusion. PET/CT imaging revealed a large, mixed-density soft tissue mass in the left lung with necrosis and indistinct boundaries, measuring approximately 97 × 157 mm at its largest dimension. The mass exhibited slightly increased heterogeneous FDG uptake, with an SUV_max_ of 2.3 (red arrow, **D**), while no FDG uptake was observed in the low-density necrotic area (green arrow, **D–F**). The contrast-enhanced CT scan showed an irregular, large soft tissue mass in the left lung with heterogeneous density and visible necrosis. The enhanced scan demonstrated heterogeneous enhancement with prominent vascular shadows (red arrow, **C**) and local encapsulation.

An 80-year-old woman presented to the hospital on May 22, 2024, with a one-month history of persistent cough and two days of chest pain. She had initially developed a dry cough one month prior, occasionally producing blood-streaked sputum, which she had dismissed as non-serious. Two days before admission, she began experiencing left-sided chest pain that she reported was not clearly related to breathing or coughing movements. The patient had no history of chronic disease and a history of other types of tumor. For further evaluation and management, she was referred to Jiangxi Provincial People’s Hospital. Contrast-enhanced CT imaging revealed a large, irregular soft tissue mass in the left lung demonstrating heterogeneous density with visible necrotic areas, measuring approximately 153 × 99 mm in maximum cross-sectional dimension. The contrast-enhanced scan showed heterogeneous enhancement with prominent vascular shadows and partial encapsulation of the lesion. These CT findings were strongly suggestive of a malignant pulmonary tumor. Tumor marker examination results included: AFP 2.9 (0–7 ng/mL), CEA 2.03 (0–6.5 ng/mL), ferritin 248.00 (30–400 ng/mL), CA199 9.16 (0–27 U/mL), NSE 21.90 (0–16.3 ng/mL), CF21-1 4.42 (0–3.3 ng/mL), CA125 104.00 (0–35 U/mL). PET/CT imaging demonstrated a large, heterogeneous soft tissue mass in the left lung measuring approximately 97 × 157 mm in maximal dimension, featuring areas of necrosis and poorly defined margins. The lesion exhibited mildly increased heterogeneous FDG uptake with a maximum standardized uptake value (SUV_max_) of 2.3. Whole-body PET/CT showed no evidence of metastatic disease. Given the relatively low metabolic activity observed, the imaging findings were most consistent with a benign solitary fibrous tumor of the lung. After multidisciplinary tumor board review, the patient proceeded to surgical resection. Histopathological examination of the resected specimen confirmed the diagnosis of solitary fibrous tumor (see Figure 2).

### 3.3 Case 3

**Figure 3:**
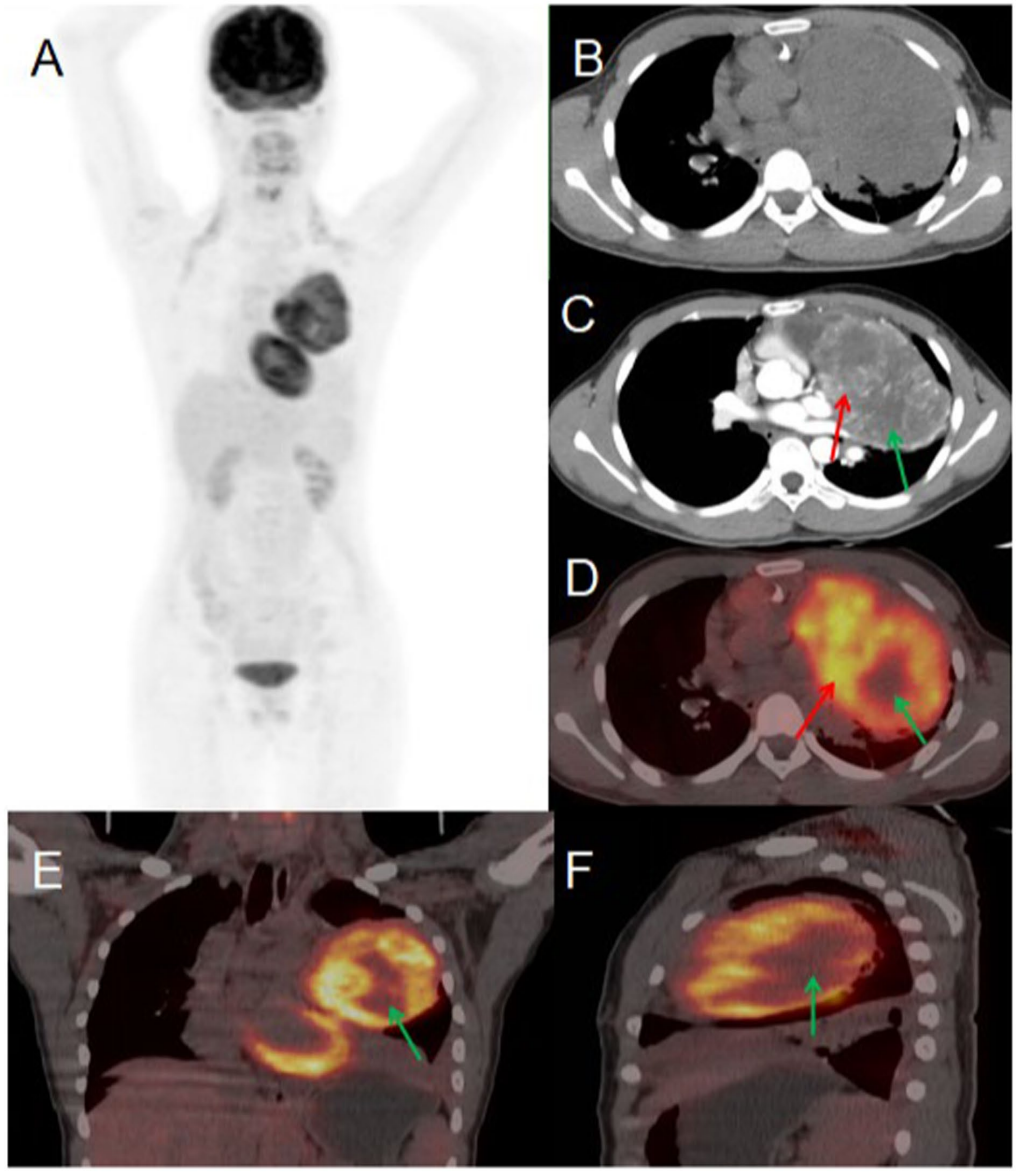
Female, 56 years old, malignant primary pulmonary solitary fibrous tumors. **(A)** Whole body MIP. **(B)** Axial, non-contrast-enhanced CT. **(C)** Axial, enhanced CT-arterial phase. **(D)** Axial fusion. **(E)** Coronal fusion. **(F)** Sagittal fusion. Contrast-enhanced CT findings: an approximately 81 × 101 mm soft tissue mass was identified in the left upper lobe, demonstrating: heterogeneous but significant enhancement in the solid components (red arrow, **C**). Non-enhancing areas corresponding to necrosis and cystic degeneration (green arrow, **C**). ^18^F-FDG PET/CT findings: the left upper lobe mass displayed: prominent necrotic-cystic degeneration throughout the lesion. Markedly increased FDG avidity in the solid components (SUV_max_ 9.6; red arrow, **D**). Absence of significant FDG uptake in the necrotic-cystic areas (green arrow, **D–F**).

A 56-year-old male presented to Jiangxi Provincial People’s Hospital on January 20, 2023, reporting a 20-day history of spontaneous left-sided chest tightness and pain unrelated to physical exertion. The symptoms had resolved spontaneously prior to presentation. The patient denied associated symptoms including hemoptysis, blood-streaked sputum, fever, night sweats, fatigue, palpitations, additional chest pain, headache, dizziness, nausea, vomiting, diarrhea, or melena. The patient had no history of chronic disease and a history of other types of tumor. He sought further evaluation and treatment at our institution. Tumor marker testing yielded the following results: NSE 19.90 (0–16.3) ng/ml, CF21-1 4.52 (0–3.3 ng/mL), AFP 6.5 (0–7 ng/mL), CEA 3.73 (0–6.5 ng/mL), Ferritin 136.00 (30–400 ng/mL), carbohydrate antigen CA199 2.75 (0–27 U/mL), CA50 0.50 (0–25 IU/mL). Contrast-enhanced CT revealed an approximately 81 × 101 mm soft tissue mass in the left upper lobe containing necrotic and cystic degenerative changes. The lesion demonstrated heterogeneous but marked enhancement, with non-enhancing necrotic and cystic components, findings suggestive of malignancy. PET/CT imaging showed the mass with significantly increased FDG uptake (SUV_max_ 9.6), supporting the preoperative diagnosis of a malignant lesion. Given the high metabolic activity (indicative of aggressive biological behavior) and absence of distant metastases on PET/CT, the multidisciplinary tumor board recommended surgical resection. Final pathological examination confirmed the diagnosis of a malignant solitary fibrous tumor of the lung (see Figure 3).

### 3.4 Case 4

**Figure 4:**
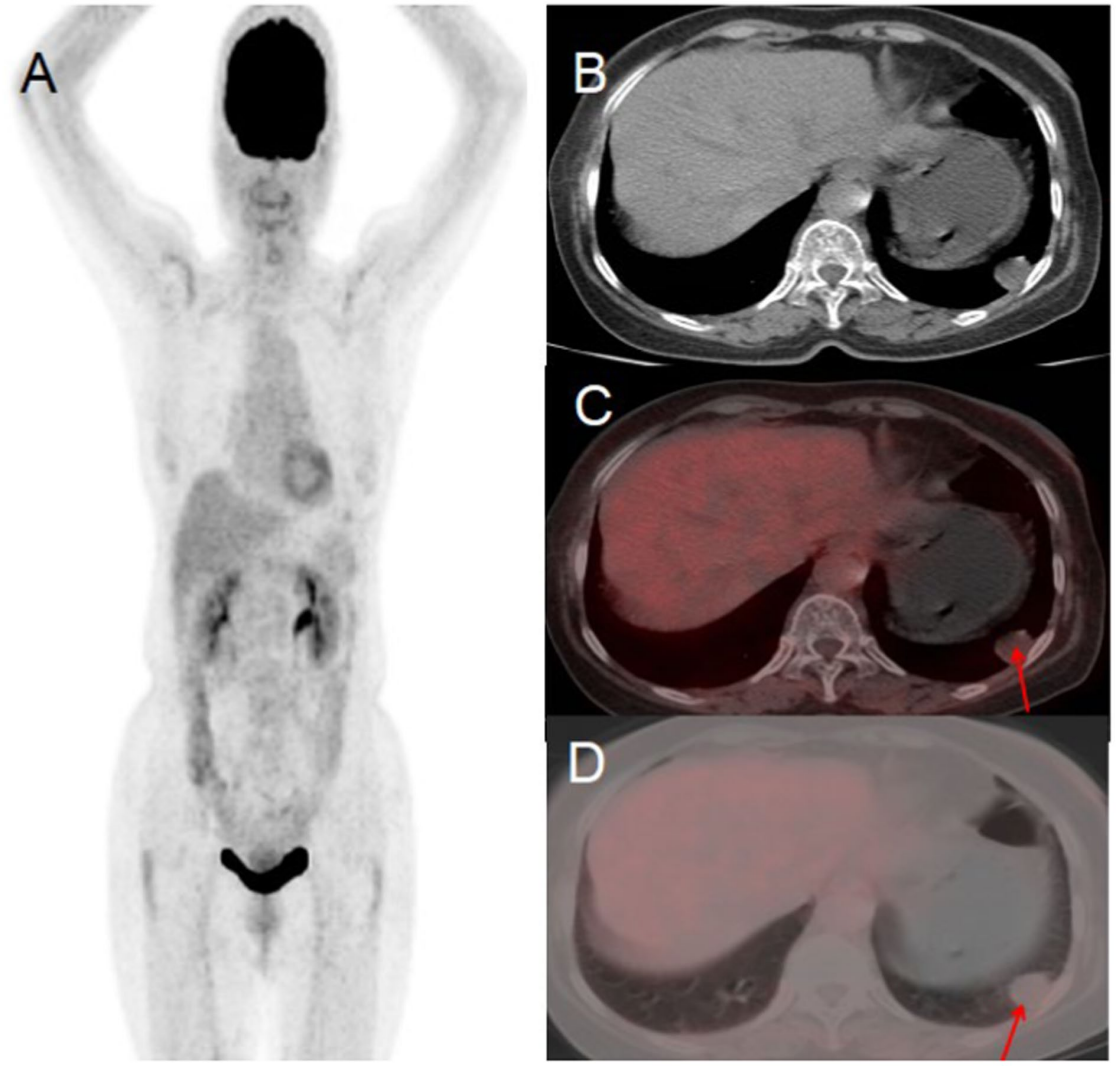
Female, 76 years old, benign primary pulmonary solitary fibrous tumors. **(A)** Whole body MIP. **(B)** Axial CT. **(C)** Axial fusion. **(D)** Axial fusion. PET/CT suggested a round lesion in the left lower lobe with mild FDG uptake, comparable to the background FDG uptake of the adjacent lung tissue (arrow, **D**).

A 76-year-old woman presented to the hospital on November 10, 2022, with a 10-day history of persistent chest tightness. She denied experiencing chest pain, hemoptysis, blood-streaked sputum, fever, night sweats, fatigue, palpitations, heart flutter, chest discomfort, headache, dizziness, nausea, vomiting, diarrhea, or black stools, the patient had no history of chronic disease and a history of other types of tumor. For further evaluation and management, she was admitted to Jiangxi Provincial People’s Hospital. Her tumor markers were all within the normal range. Chest CT revealed a round, soft-tissue-density lesion in the left lower lobe of the lung, which showed mild enhancement on contrast imaging, suggestive of a benign tumor. PET/CT demonstrated a round lesion in the left lower lobe with mild FDG uptake, similar to the background FDG uptake of the adjacent lung tissue. Based on the PET/CT findings, the preoperative diagnosis was a benign lung tumor. After a multidisciplinary team discussion, surgical resection was recommended. Postoperative pathology confirmed a benign solitary fibrous tumor of the lung (see Figure 4).

### 3.5 Findings summary

The four patients exhibited diverse clinical manifestations. Three presented with chest tightness and pain, while one had chest tightness without pain. Two patients reported cough, but none experienced hemoptysis, sputum production, or fever. Contrast-enhanced CT classified three cases as malignant and one as benign. On ^18^F-FDG PET/CT, two cases demonstrated high FDG uptake and were preoperatively diagnosed as malignant—one with abdominal metastasis and the other without. The remaining two cases showed low FDG uptake and were diagnosed as benign, with no evidence of metastasis. All diagnoses were surgically confirmed. Compared to pathological findings, preoperative PET/CT achieved 100% accuracy in differentiating benign from malignant primary solitary fibrous tumors of the lung, whereas contrast-enhanced CT had only 50% accuracy, underscoring the diagnostic superiority of PET/CT. Furthermore, PET/CT proved critical for guiding percutaneous biopsy in Case 1 and was invaluable in assessing post-chemotherapy tumor activity. The clinical and laboratory characteristics of these patients are summarized in Table 1.

**Table 1:** Clinical features of pulmonary solitary fibrous tumors and imaging findings of ^18^F-FDG PET/CT.

Case	Case 1	Case 2	Case 3	Case 4
Gender	Female	Male	Female	Female
Year	70	80	56	76
Thoracic oppression	Yes	Yes	Yes	Yes
Thoracic pain	Yes	Yes	Yes	No
Cough	Yes	Yes	No	No
Tumor marker (CF21-1, NSE)	Mild elevation	Mild elevation	Mild elevation	Normal
Tumor size (mm)	88 × 108 mm	97 × 157 mm	81 × 101 mm	21 × 16 mm
SUV_max_	23.2	2.0	9.6	1.4
Contrast-enhanced CT	Marked enhancement	Mild enhancement	Marked enhancement	Mild enhancement
Benign or malignant	Malignant	Benign	Malignant	Benign
Metastasis	Yes	No	No	No
Treatment method	Surgery after chemotherapy	Surgery	Surgery	Surgery
Follow-up	No recurrence	No recurrence	No recurrence	No recurrence

### 3.6 Treatment and follow-up

Case 1: Preoperative PET/CT imaging diagnosed a malignant solitary fibrous tumor of the lung with abdominal metastasis. The patient was administered bevacizumab at a dose of 400 mg on day 1, in combination with temozolomide at 220 mg on days 1–5 as part of the chemotherapy regimen. After one year of treatment, a follow-up PET/CT scan was performed to evaluate the response to therapy. The scan indicated a favorable response, with significant tumor regression and the inactivation of abdominal metastatic lesions. Subsequently, the lung lesion was surgically resected. The patient has been followed for one year postoperatively, with no recurrence observed. Case 3: The preoperative PET/CT scan revealed high FDG uptake in the lung lesion, which was diagnosed as a malignant solitary fibrous tumor. No metastasis was detected throughout the body, prompting the direct surgical resection of the lung lesion. Following surgery, the patient received comprehensive antitumor therapy and has been monitored since; there have been no signs of recurrence to date. Cases 2 and 4: Both lung lesions demonstrated mild FDG uptake on preoperative PET/CT scans, leading to a diagnosis of benign solitary fibrous tumors. Surgical resection of the lung lesions was carried out promptly. To date, follow-up has revealed no abnormalities in either case.

## 4 Discussion

Solitary fibrous tumors (SFTs) are rare soft tissue neoplasms believed to originate from CD34 antigen-expressing dendritic mesenchymal cells (5). These cells are widely distributed throughout the body’s connective tissues, suggesting that SFTs may develop in virtually any anatomical location. However, the pleura remains the most frequent site of occurrence (6), where these tumors typically differentiate into myofibroblasts and fibroblasts. While most SFTs follow a benign clinical course, approximately 18% demonstrate aggressive behavior associated with potential recurrence or metastasis (7, 8). Notably, even histologically benign SFTs may undergo malignant transformation over time, acquiring local invasive capabilities.

The pathogenesis of solitary fibrous tumors (SFTs) remains incompletely understood, though current evidence suggests a strong association with the NAB2-STAT6 fusion gene resulting from chromosomal inversion at 12q13. This genetic alteration, present in nearly all SFT cases regardless of anatomical location or histological subtype, is now recognized as a critical driver of tumorigenesis and disease progression (9). The clinical presentation of pulmonary SFTs varies considerably depending on tumor location, size, degree of cellular differentiation, and relationship to adjacent structures. These typically slow-growing masses are often discovered incidentally, though they may become symptomatic through mass effect on surrounding organs. Rarely, SFTs can cause paraneoplastic hypoglycemia, likely mediated by tumor-secreted insulin-like growth factor (IGF) (10, 11). While SFTs may occur at any age, the incidence peaks between 50–70 years, with pediatric cases being uncommon. The gender distribution shows a slight male predominance (approximately 3:2 ratio) (8, 12). Gross examination typically reveals a well-circumscribed tumor with a glistening fibrous capsule. The cut surface demonstrates a firm, gray to tan-yellow appearance, often with areas of myxoid change, hemorrhage, or necrosis. Histologically, SFTs are characterized by a haphazard proliferation of bland oval to spindle cells within a collagenous stroma showing variable myxoid degeneration (13, 14). A distinctive feature is the presence of branching, “staghorn” vascular pattern with frequent perivascular hyalinization. Malignant transformation is suggested by several pathological features: infiltrative margins, cellular pleomorphism, nuclear atypia, increased mitotic activity, and necrosis. Immunohistochemically, CD34 expression is observed in 79–100% of cases, serving as a valuable diagnostic marker (15, 16).

The imaging characteristics of solitary fibrous tumors (SFTs) of the lung demonstrate heterogeneity while maintaining certain consistent features (17). On CT imaging, pulmonary SFTs typically present as solitary lesions. Benign variants are characterized by well-circumscribed margins, with smaller lesions appearing round or oval and larger tumors exhibiting lobulated or irregular contours. Non-contrast CT reveals isodense attenuation relative to adjacent muscle tissue. MRI findings include isointense signal on T1-weighted imaging and variable (isointense to hyperintense) signal on T2-weighted sequences. Internal heterogeneity may occur secondary to cystic degeneration, necrosis, hemorrhage, or calcification (18). Both contrast-enhanced CT and MRI typically demonstrate pronounced but heterogeneous enhancement. Malignant pulmonary SFTs display aggressive features including infiltrative borders, internal density heterogeneity, and multifocal necrosis. Contrast-enhanced studies reveal hypervascularity with intratumoral abnormal vascular enhancement, sometimes exhibiting a characteristic “map-like” enhancement pattern (19). In our series, two cases showed marked enhancement suggestive of hypervascularity, while two others demonstrated mild-to-moderate enhancement corresponding to areas of hypocellularity and hyalinized collagen bundles (20). ^18^F-FDG PET/CT provides functional metabolic imaging capable of revealing tumor activity at the molecular level, serving as a valuable tool for tumor characterization and staging. Whole-body imaging allows comprehensive evaluation of both primary lesions and distant sites. Malignant SFTs typically demonstrate heterogeneous ^18^F-FDG uptake with photopenic necrotic regions (21). Traditionally, differentiation between benign and malignant pulmonary SFTs relied on CT features and clinical presentation, though this remains diagnostically challenging. Previous studies suggest malignant potential is associated with lesions exceeding 10 cm, particularly those demonstrating cystic or cystic-solid components (22). The presence of “geographic” enhancement with delayed contrast uptake on CT has also been strongly associated with malignancy (23). However, these imaging criteria are not absolute. A notable exception in our series was Case 3, where a massive tumor (>10 cm) with extensive necrosis and heterogeneous “geographic” enhancement on contrast CT—features typically predictive of malignancy—was pathologically confirmed as benign, challenging conventional imaging paradigms. Our findings suggest PET/CT may offer superior diagnostic accuracy in differentiating benign from malignant pulmonary SFTs. Malignant lesions consistently demonstrated heterogeneously increased ^18^F-FDG uptake with non-avid necrotic regions, while benign lesions showed low FDG avidity. Remarkably, PET/CT achieved 100% diagnostic accuracy in preoperative classification, corroborating previous reports regarding the utility of SUV_max_ measurements (24). Zhao et al. (25) similarly found significantly higher metabolic activity in malignant versus benign pulmonary SFTs. These observations align with Gorospe’s (26) report of a benign SFT case demonstrating SUV_max_ of 1.8 (below the conventional diagnostic threshold of 2.5), with subsequent pathological confirmation. However, our series also identified an atypical presentation in Case 1, which showed intense primary tumor avidity (SUV_max_ 13.2) along with FDG-avid nodular lesions near the pancreatic head and body, radiologically suggestive of peritoneal metastases—a finding discordant with established literature. These results indicate that ^18^F-FDG PET/CT not only facilitates noninvasive differentiation of benign and malignant pulmonary SFTs but also enables detection of systemic metastases (27). This modality provides comprehensive staging guidance, optimal biopsy site selection, and valuable diagnostic integration with clinical and laboratory findings.

The clinical and imaging presentations of solitary fibrous tumors (SFTs) of the lung often mimic those of other conditions such as fibrohistiocytomas, fibrosarcomas, and synovial sarcomas. While ^18^F-FDG PET/CT is a valuable diagnostic tool, it alone cannot reliably differentiate SFTs from these other entities (28), making pathological confirmation essential for definitive diagnosis. For benign pulmonary SFTs, surgical resection remains the treatment of choice (29). However, the management of malignant pulmonary SFTs lacks consensus, with extensive surgical resection being the primary approach (30). Emerging evidence suggests that patients may benefit from metastasectomy when the primary tumor has been completely resected (31). For metastatic or unresectable cases, chemotherapy regimens have been employed, though their efficacy remains uncertain (32). In Case 1 of our series, the patient presented with malignant pulmonary SFTs and intraperitoneal metastasis. Following multidisciplinary evaluation, neoadjuvant chemotherapy was initiated to reduce tumor burden prior to surgical intervention. The treatment regimen consisted of bevacizumab (400 mg on day 1) combined with temozolomide (220 mg on days 1–5) in the first cycle, followed by cindilizumab (200 mg) plus anlotinib (12 mg on days 1–14). Serial ^18^F-FDG PET/CT imaging after one year of therapy demonstrated significant reduction in both size and metabolic activity of the primary pulmonary lesion, along with complete suppression of abdominal metastatic activity. Based on these favorable imaging findings, the patient subsequently underwent successful surgical resection with excellent postoperative recovery. Our experience suggests that for malignant SFTs, ^18^F-FDG PET/CT should first be utilized to evaluate tumor characteristics, including benign/malignant potential and metastatic status, with particular attention to signs of local tissue infiltration. This comprehensive assessment can guide therapeutic decision-making. In Case 1, the combination of bevacizumab and temozolomide followed by surgical resection yielded promising results. The remaining three cases in our series, after PET/CT confirmation of no distant metastases, underwent immediate surgical resection with similarly favorable outcomes. The clinical course of SFTs exhibits considerable variability, with reported median survival ranging from 5 to 94 months. The 5-year and 10-year survival rates are approximately 89 and 73%, respectively (33). Notably, even with negative surgical margins, these tumors may recur locally or metastasize to various sites including lung, liver, adrenal glands, bones, brain, muscles, and gastrointestinal tract. Metastatic disease portends a poor prognosis, with 75% of affected patients demonstrating median survival of only 22–46 months (34, 35). The unpredictable biological behavior of SFTs poses significant challenges for prognostication. Histologically benign-appearing tumors may demonstrate aggressive clinical behavior, while some histologically atypical tumors may follow an indolent course (36). Furthermore, tumors initially classified as low-grade may exhibit malignant transformation upon recurrence or metastasis (37). Although most recurrences manifest within 2 years postoperatively, late recurrences up to 17 years after initial treatment have been documented (38, 39). Given this unpredictable natural history, we recommend close surveillance with CT imaging every six months for the first two postoperative years. While ^18^F-FDG PET/CT is more costly, its superior sensitivity justifies annual performance, particularly in the follow-up of malignant pulmonary SFTs where it can provide valuable prognostic information.

Pulmonary SFTs represent a rare subset of mesenchymal-derived soft-tissue neoplasms, exhibiting infrequent clinical occurrence and often atypical imaging manifestations. Histopathological and immunohistochemical analyses remain indispensable for definitive diagnosis. Notably, ^18^F-FDG PET/CT imaging reveals distinctive features in pulmonary SFTs, including heterogeneously increased FDG avidity with photopenic regions corresponding to necrotic components. These characteristics underscore the potential utility of preoperative ^18^F-FDG PET/CT in predicting the biological behavior (benign vs. malignant) of pulmonary SFTs, thereby significantly guiding clinical decision-making. Furthermore, for histologically confirmed malignant pulmonary SFTs, ^18^F-FDG PET/CT demonstrates substantial clinical value in monitoring therapeutic response.

## Data Availability

The datasets presented in this study can be found in online repositories. The names of the repository/repositories and accession number(s) can be found in the article/supplementary material.
